# How low can dietary greenhouse gas emissions be reduced without impairing nutritional adequacy, affordability and acceptability of the diet? A modelling study to guide sustainable food choices

**DOI:** 10.1017/S1368980016000653

**Published:** 2016-04-06

**Authors:** Marlène Perignon, Gabriel Masset, Gaël Ferrari, Tangui Barré, Florent Vieux, Matthieu Maillot, Marie-Josèphe Amiot, Nicole Darmon

**Affiliations:** 1 NORT, Aix-Marseille Université, INRA, INSERM, Faculté de Médecine de la Timone, 27 Boulevard Jean Moulin, 13005 Marseille, France; 2 MS-Nutrition, Marseille, France

**Keywords:** Diet-related greenhouse gas emissions, Diet sustainability, Food choices, Linear programming modelling, Food consumption, Dietary changes, Diet cost, Nutritional quality, Affordability, Cultural acceptability

## Abstract

**Objective:**

To assess the compatibility between reduction of diet-related greenhouse gas emissions (GHGE) and nutritional adequacy, acceptability and affordability dimensions of diet sustainability.

**Design:**

Dietary intake, nutritional composition, GHGE and prices were combined for 402 foods selected among those most consumed by participants of the Individual National Study on Food Consumption. Linear programming was used to model diets with stepwise GHGE reductions, minimized departure from observed diet and three scenarios of nutritional constraints: none (FREE), on macronutrients (MACRO) and for all nutrient recommendations (ADEQ). Nutritional quality was assessed using the mean adequacy ratio (MAR) and solid energy density (SED).

**Setting:**

France.

**Subjects:**

Adults (*n* 1899).

**Results:**

In FREE and MACRO scenarios, imposing up to 30 % GHGE reduction did not affect the MAR, SED and food group pattern of the observed diet, but required substitutions within food groups; higher GHGE reductions decreased diet cost, but also nutritional quality, even with constraints on macronutrients. Imposing all nutritional recommendations (ADEQ) increased the fruits and vegetables quantity, reduced SED and slightly increased diet cost without additional modifications induced by the GHGE constraint up to 30 % reduction; higher GHGE reductions decreased diet cost but required non-trivial dietary shifts from the observed diet. Not all the nutritional recommendations could be met for GHGE reductions ≥70 %.

**Conclusions:**

Moderate GHGE reductions (≤30 %) were compatible with nutritional adequacy and affordability without adding major food group shifts to those induced by nutritional recommendations. Higher GHGE reductions either impaired nutritional quality, even when macronutrient recommendations were imposed, or required non-trivial dietary shifts compromising acceptability to reach nutritional adequacy.

Sustainable diets are defined as nutritionally adequate, safe and healthy, culturally acceptable, financially affordable and with low environmental impacts^(^ ^1^ ^)^. Reducing emissions arising from the food system is a major lever for achieving greenhouse gas emission (GHGE) reduction targets^(^ ^2^ ^–^ ^4^ ^)^. This reduction could be partly achieved through shifts in food consumption since the same quantities of different foods emit different levels of greenhouse gas, with livestock products being the largest emitters^(^ ^5^ ^)^. In the pioneering report *How Low Can We Go?*, Audsley *et al.* ^(^ ^6^ ^)^ explored a set of scenarios to reduce UK GHGE by 70 % by 2050, and suggested that a vegetarian diet or a reduction in livestock product consumption could help meet this target. In parallel, public health authorities recommend consuming plenty of plant-based foods and a moderate amount of animal products, especially red and processed meats^(^ ^7^ ^)^. Hence it has been suggested that dietary changes aimed at reducing diet-related GHGE may also promote health^(^ ^8^ ^,^ ^9^ ^)^.

Some studies investigating dietary scenarios^(^ ^10^ ^,^ ^11^ ^)^ or self-selected diets^(^ ^12^ ^)^ with reduced meat consumption in the UK have strengthened the message of a compatibility between health and environmental dimensions. However, in a Dutch cohort, the GHGE of usual diets were not associated with mortality, indicating that an environmentally friendlier diet is not necessarily a healthier diet^(^ ^13^ ^)^. Other studies showed that higher nutritional quality was associated with higher GHGE in self-selected French diets^(^ ^14^ ^,^ ^15^ ^)^. Thus there is still no consensus on the compatibility between health or nutrition and environmental dimensions.

Affordability is another important dimension of sustainable diets^(^ ^1^ ^)^. Healthy diets are known to be generally more expensive than unhealthy diets^(^ ^16^ ^,^ ^17^ ^)^, as recently confirmed in studies investigating affordability, healthy dietary pattern and GHGE^(^ ^12^ ^)^.

Diet optimization by linear programming has been used in human nutrition to assess the compatibility between nutritional adequacy and affordability of diets, or the gaps in consumption between observed and recommended dietary patterns at population and individual levels^(^ ^18^ ^–^ ^21^ ^)^. Linear programming is also a powerful tool to design nutritious diets with a low environmental impact: two studies combined affordability, GHGE reduction and nutritional adequacy in linear programming models^(^ ^22^ ^,^ ^23^ ^)^. However, these modelling studies were not based on observed food consumption data, which may compromise the cultural acceptability of the proposed modelled diets.

The main objective of the present study was to assess – using diet modelling with linear programming – the compatibility between the reduction of dietary GHGE and the other dimensions of diet sustainability, namely nutritional adequacy, cultural acceptability and affordability. The population’s mean observed diet was considered a proxy for a culturally acceptable diet. Thus, to avoid deteriorating the acceptability as much as possible, the models minimized the departure, in terms of food content, from the mean observed diet. The impact of stepwise GHGE reductions on nutritional quality, cost and cultural acceptability of modelled diets was assessed for increasingly stringent levels of nutritional constraints.

## Materials and methods

### Population sample and dietary data

Dietary intakes were derived from the 7 d food records of a nationally representative stratified random sample of French adults (*n* 2624; aged >18 years) participating in the Second French Individual and National Study on Food Consumption cross-sectional dietary survey (*Étude Individuelle et Nationale sur les Consommations Alimentaires*, INCA2), conducted in 2006–2007 by ANSES (the French agency for food, environmental and occupational health safety)^(^ ^24^ ^)^. After exclusion of energy under-reporters using Black’s equations^(^ ^25^ ^)^ and individuals consuming hypo-energetic meal substitutes, the present analysis was conducted on a final sample of 1899 adults, aged 47·1 (sd 15·3) years, of whom 1126 were women. The CIQUAL database associated with the survey gives the detailed nutrient composition of all the foods declared to be consumed by the participants (1342 foods and beverages, including water).

### Greenhouse gas emissions of foods

GHGE estimates, expressed in grams of CO_2_ equivalents (g eqCO_2_), were assigned by the environmental consulting firm Bio by Deloitte (formerly Greenext Service, Paris, France) to 402 foods either selected as being among those most consumed by INCA2 participants, as described by Vieux *et al.* ^(^ ^14^ ^)^, or identified as having a potential nutritional and/or environmental utility (e.g. soya-based products, some unrefined starchy foods, chestnuts). The GHGE values were assigned based on a hybrid input–output/life cycle assessment (LCA) method using the international ISO 1404x:2006 LCA standards^(^ ^26^ ^,^ ^27^ ^)^ and the specific French BP X30-323-0 guidelines^(^ ^28^ ^)^. The estimates include the whole life cycle of foods, from farm production to usage and waste management of packaging, but exclude emissions arising from indirect land-use change and the highly variable emissions from consumers’ transport from retail to home. For composite food items, the LCA analysis considers the GHGE of each ingredient and their proportion in the product, based on the recipes provided by the INCA2 survey. The hybrid approach combined French trade and production data^(^ ^29^ ^,^ ^30^ ^)^ and standard life cycle inventory data (e.g. Ecoinvent^(^ ^31^ ^)^) so that the GHGE values assigned reflected the average food products as consumed in the French market^(^ ^32^ ^)^.

### Aggregation of dietary data

The intakes of the 1342 foods declared to be consumed by INCA2 participants were aggregated into the 402 foods for which GHGE estimates were available, using a nutritional Euclidean distance method developed specifically for the study (see online supplementary material, Supplemental methods, for a full description). Energy and nutrient intakes calculated with the original database of 1342 foods and with the aggregated database of 402 foods are given in Supplemental Table 1.

### Diet modelling by linear programming

Linear programming models were developed to design diets with reduced GHGE and subjected to a set of nutritional constraints, while remaining as close as possible to the mean diet of the French adult population. The impact of the GHGE constraint on food choices, nutritional quality and cultural acceptability was evaluated by incrementally decreasing diet-related GHGE. Three levels of nutritional exigencies were defined by increasingly stringent nutritional constraints. The cultural acceptability dimension was considered through the objective function by minimizing departure from the mean observed diet. In addition, acceptability constraints on food quantities and energy were used in all models to ensure that the modelled diets remained within the range of diets actually consumed by the general French adult population. All linear programming models were run using the statistical software package SAS version 9·4. The characteristics of the linear programming models (objective function and constraints) are summarized in Table 1.

**Table 1:** Constraints in the FREE, MACRO and ADEQ scenarios

	Women	Men	FREE	MACRO	ADEQ	Reference
Nutritional constraints						
Proteins (% of total energy)	10–20		–	applied	applied	^(^ ^33^ ^)^
Carbohydrates (% of total energy)	50–75		–	applied	applied	^(^ ^34^ ^)^
Fats (% of total energy)	20–35		–	applied	applied	^(^ ^35^ ^)^
Linolenic acid (% of total energy)	>0·5		–	–	applied	^(^ ^35^ ^)^
Linoleic acid (% of total energy)	2·5–9·0		–	–	applied	^(^ ^35^ ^)^
EPA+DHA (g/d)	>0·25		–	–	applied	^(^ ^35^ ^)^
PUFA (% of total energy)	6–11		–	–	applied	^(^ ^35^ ^)^
SFA (% of total energy)	<10		–	–	applied	^(^ ^7^ ^)^
Cholesterol (mg/d)	<300		–	–	applied	^(^ ^7^ ^)^
Free sugars (% of total energy)	<10		–	–	applied	^(^ ^7^ ^)^
Na (mg/d)	1500–2365	1500–2759	–	–	applied	^(^ ^37^ ^)^
Fibre (g/d)	>30	>30	–	–	applied	^(^ ^36^ ^)^
Ca (mg/d)	≥900	≥900	–	–	applied	^(^ ^36^ ^)^
Cu (mg/d)	≥1·5	≥2	–	–	applied	^(^ ^36^ ^)^
Fe (mg/d)	≥16	≥9	–	–	applied	^(^ ^36^ ^)^
Iodine (mg/d)	≥150	≥150	–	–	applied	^(^ ^36^ ^)^
Mg (mg/d)	≥360	≥420	–	–	applied	^(^ ^36^ ^)^
P (mg/d)	≥750	≥750	–	–	applied	^(^ ^36^ ^)^
K (mg/d)	≥3100	≥3100	–	–	applied	^(^ ^36^ ^)^
Se (µg/d)	≥50	≥60	–	–	applied	^(^ ^36^ ^)^
Vitamin A (µg/d)	600–1600	800–1800	–	–	applied	^(^ ^36^ ^)^
Thiamin (mg/d)	≥1·1	≥1·3	–	–	applied	^(^ ^7^ ^)^
Riboflavin (mg/d)	≥1·5	≥1·6	–	–	applied	^(^ ^7^ ^)^
Niacin (mg/d)	≥11	≥14	–	–	applied	^(^ ^7^ ^)^
Vitamin B_12_ (µg/d)	≥2·4	≥2·4	–	–	applied	^(^ ^7^ ^)^
Pantothenic acid (mg/d)	≥5	≥5	–	–	applied	^(^ ^7^ ^)^
Vitamin B_6_ (mg/d)	≥1·5	≥1·8	–	–	applied	^(^ ^7^ ^)^
Folic acid (µg/d)	≥300	≥330	–	–	applied	^(^ ^7^ ^)^
Vitamin C (mg/d)	≥110	≥110	–	–	applied	^(^ ^7^ ^)^
Vitamin D (µg/d)	≥5	≥5	–	–	applied	^(^ ^7^ ^)^
Vitamin E (mg/d)	≥12	≥12	–	–	applied	^(^ ^7^ ^)^
Zn (mg/d)	≥10	≥12	–	–	applied	^(^ ^7^ ^)^
Total energy (kcal/d)	Equal to the total energy of the mean observed diet*		applied	applied	applied	
Cultural acceptability constraints						
Total weight	80–120 % of total weight of the mean observed diet†		applied	applied	applied	
Foods, food groups and subgroups	<90th percentile, calculated on the mean observed diet†,‡		applied	applied	applied	
Environmental constraints						
Total dietary GHGE	Incremental reduction (10 %) from level in the observed diet		applied	applied	applied	

FREE; no nutritional constraints; MACRO, constraints on macronutrients only; ADEQ, constraints on all nutrients; GHGE, greenhouse gas emissions.

* 8109 kJ/d (1938 kcal/d) for women, 10 891 kJ/d (2603 kcal/d) for men.

† Calculated for men and women separately.

‡ For foods, non-consumers excluded; for food subgroups and groups, non-consumers included.

#### Nutritional constraints defining the FREE, MACRO and ADEQ scenarios

To assess the compatibility between the imposed reductions of dietary GHGE and nutritional quality of modelled diets, three nutritional scenarios were designed based on increasingly stringent levels of nutritional constraints: (i) no nutritional constraints (FREE scenario); (ii) constraints on macronutrients only (MACRO scenario), in which proteins, total fat and carbohydrates were constrained to minimal and maximal percentages of total energy; and (iii) constraints on all nutrients (ADEQ scenario), in which, in addition to macronutrients, micronutrients, fibre and fatty acids were constrained to a minimal quantity based on the RDA estimated to meet the nutrient needs of 97·5 % of adults in the population and/or a maximal quantity based on recommended upper limits. The values of the nutritional constraints, summarized in Table 1, were derived from WHO recommendations for proteins^(^ ^7^ ^,^ ^33^ ^)^, carbohydrates^(^ ^34^ ^)^, total fat, linoleic acid, α-linolenic acid, DHA and EPA, total PUFA^(^ ^35^ ^)^, cholesterol, SFA and free sugars^(^ ^7^ ^)^; from the French recommendations for fibre, ten vitamins and nine minerals^(^ ^36^ ^)^; and from the Nordic Nutrient Recommendations for Na^(^ ^37^ ^)^. The models were run separately for men and women since observed intakes and nutritional recommendations differ between genders.

#### Environmental constraint: reductions in greenhouse gas emissions

For each of the three scenarios of nutritional constraints, modelled diets were designed at increasingly stringent levels of GHGE reduction. The constraint imposing the total dietary GHGE reduction was gradually strengthened in 10 % steps, starting from no imposed reduction from the GHGE value of the mean observed diet up to the maximal reduction achievable.

#### Acceptability constraints

To avoid unrealistic modelled diets, the total food quantity was constrained to range between 80 % and 120 % of the mean observed intake (2920 g/d for men, 2581 g/d for women); the total energy had to be equal to the energy of the mean observed diet (10 916 kJ/d (2609 kcal/d) for men and 8109 kJ/d (1938 kcal/d) for women); and the food item, food group and food subgroup quantities were constrained to be lower than the 90th percentile of the observed intakes. The percentiles were calculated by gender, for consumers only in the case of food items and for the whole population in the case of food groups and subgroups.

#### Objective function

In order to translate the objective of remaining as close as possible to the observed diet, the objective function of the linear programming models was defined as the minimization of the total departure between the observed and modelled diets, at both the food item (*n* 402) and the food group (*n* 8; adapted from the food groups used for the French food-based dietary guidelines^(^ ^38^ ^)^: Meat/Fish/Eggs, Dairy Products, Fruit and Vegetables, Starch, Foods High in Fat/Salt/Sugar, Drinks, Seasonings, Mixed Dishes) levels. The objective function was expressed mathematically by:
1

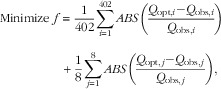

where *i* represents the 402 foods and *j* the eight food groups, *ABS* refers to absolute value, *Q*_obs_ is the mean observed quantity and *Q*_opt_ the optimized quantity.

The absolute function being non-linear, *f* was then transformed into a linear function, as previously described by Darmon *et al.* ^(^ ^19^ ^)^, so that linear programming could be applied.

An alternative objective function minimizing the GHGE was used to assess the maximal GHGE reduction achievable for each scenario.

#### Strength of nutritional constraints

The dual value, calculated for each nutritional constraint as the improvement in the objective function when the constraint is relaxed by 1 %, enabled us to evaluate how restrictive the constraints were and to compare their strength. A non-zero dual value indicated that the corresponding constraint was restrictive, i.e. that fulfilling the constraint had an influence on food selection and thus on deviation from the observed diet. A null dual value indicated that the constraint was not restrictive. The higher the absolute dual value, the more difficult the constraint was to meet.

### Nutritional quality assessment

The mean adequacy ratio (MAR), the mean excess ratio (MER) and the solid energy density (SED) were used to assess the nutritional quality of the observed and modelled diets, as previously described by Vieux *et al.* ^(^ ^14^ ^)^.

The MAR was calculated for each diet as the mean percentage of daily recommended intakes for twenty key nutrients (proteins, fibre, Ca, K, Fe, Mg, Zn, Cu, iodine, Se, vitamin A, vitamin C, vitamin D, vitamin E, thiamin, riboflavin, niacin, vitamin B_6_, folic acid and vitamin B_12_) by:
2

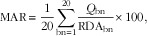

where *Q*_bn_ is the daily quantity of each beneficial nutrient (bn) and RDA_bn_ is the corresponding recommended intake for this nutrient. The reference values for the twenty recommended nutrients are given in Table 1. Each ratio (*Q*_bn_/RDA_bn_×100)>100 was set to 100, so that a high intake of one nutrient could not compensate for the low intake of another^(^ ^14^ ^)^.

MER was calculated for each diet as the mean daily percentage of the maximum recommended values (MRV) for three nutrients to limit, namely SFA, Na and free sugars, by:
3

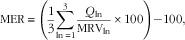

where *Q*_ln_ is the daily quantity of each nutrient to limit (ln) and MRV_ln_ is the corresponding maximum recommended value for this nutrient (Table 1). The term ‘free sugars’ refers to added sugars plus sugars naturally present in honey, syrups and fruit juices^(^ ^7^ ^)^. The MRV for SFA and free sugars corresponded to 10 % of the energy of the mean observed diet. The MRV for Na was 2759 and 2365 mg for men and women, respectively, and corresponded to a daily intake of 7 and 6 g NaCl. Each ratio (*Q*_ln_/MRV_ln_×100) <100 was set to 100, so that a low intake of one harmful nutrient could not compensate for the high intake of another.

The SED, expressed in kcal/100 g (1 kcal=4·184 kJ), was calculated as the ratio between energy intake and diet weight. As proposed by Ledikwe *et al*.^(^ ^39^ ^)^, only items typically consumed as foods, including soups, were included in the calculation of SED, whereas foods typically consumed as beverages, such as milk, juices and other drinks, were excluded.

### Acceptability assessment

The dimension of acceptability was assessed by analysing the changes of quantity (g/d) occurring for each food group and subgroup, and by calculating the percentage of departure from food quantities in the population’s mean observed diet. It was assumed that diets similar, in terms of food composition, to the mean observed diet could be considered as culturally acceptable, with the greatest departure from the observed diet having the highest risk of lower cultural acceptability. The absolute departure was calculated both at the food item level, corresponding to the first term of the objective function, and at the food group level, corresponding to the second term of the objective function.

### Diet cost assessment

Diet cost was estimated based on food prices obtained from the 2006 Kantar Worldpanel purchase database^(^ ^40^ ^)^, which gives the annual expenditure and the quantity purchased for each food item available on the market in a representative sample of 12000 French households. The mean national prices of the 402 food items were calculated by dividing annual expenditures by the quantities purchased, as previously described by Masset *et al.* ^(^ ^15^ ^)^, and are representative of the most frequently purchased form of each item.

## Results

The GHGE of the mean observed diet were 3667 g eqCO_2_/d and 4896 g eqCO_2_/d for women and men, respectively. The maximal GHGE reductions from these observed levels achievable under the constraints were 82·6 %, 82·2 % and 69·7 % reduction for women, and 81·9 %, 79·9 % and 74·0 % reduction for men, for the FREE, MACRO and ADEQ scenarios, respectively.

Results of the diets modelled by linear programming are detailed below for women’s models only. Results obtained for men’s models are presented in the online supplementary material, Supplemental Table 2 and Supplemental Figs 1–3.

### Impact of reduction in greenhouse gas emissions on nutritional quality

**Fig. 1:**
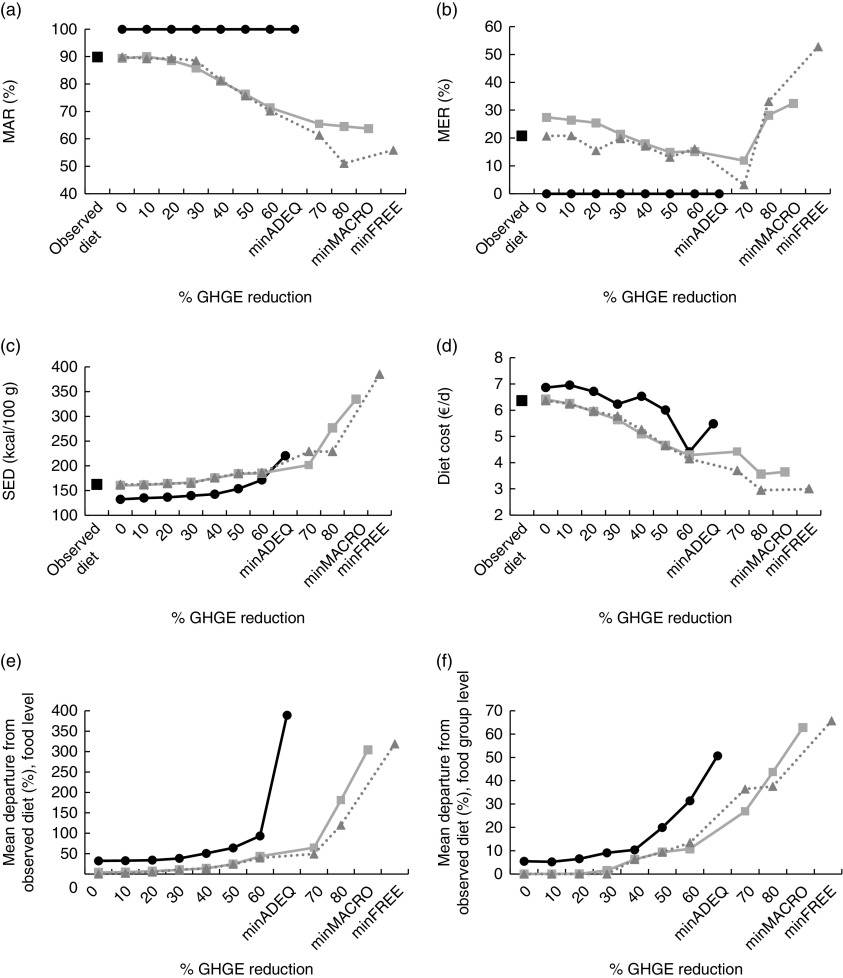
(a) Mean adequacy ratio (MAR), (b) mean excess ratio (MER), (c) solid energy density (SED), (d) diet cost, (e) mean departure from the observed diet at food level and (f) mean departure from the observed diet at food group level, for the mean observed diet (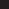) and for modelled diets at different levels of dietary GHGE reduction under the FREE (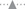), MACRO (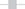) and ADEQ (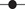) scenario, for French women (GHGE, greenhouse gas emissions; FREE; no nutritional constraints; MACRO, constraints on macronutrients only; ADEQ, constraints on all nutrients; minFREE, maximal GHGE reduction (82·6 %) achievable under the constraints of the FREE scenario; minMACRO, maximal GHGE reduction (82·2 %) achievable under the constraints of the MACRO scenario; minADEQ, maximal GHGE reduction (69·7 %) achievable under the constraints of the ADEQ scenario; 1 kcal=4·184 kJ)

The MAR, MER and SED of the observed and modelled diets are presented in Fig. 1(a) to 1(c).

In the mean observed diet, the MAR, MER and SED were 89·9 %, 20·7 % and 162 kcal/100 g (678 kJ/100 g), respectively. Thirteen nutrients had a content below 100 % of the RDA, with fibre, vitamin D and Fe contents in particular covering less than 80 % of the RDA (Table 2). SFA and Na contents exceeded the recommended upper limits.

**Table 2:** Nutrient levels (percentage of the RDA or percentage of the upper limit) in the mean observed diet and in the FREE, MACRO and ADEQ modelled diets at different levels of GHGE reduction from the value of the mean observed diet for French women

		FREE										MACRO										ADEQ							
	Observed	% GHGE reduction										% GHGE reduction										% GHGE reduction							
Nutrient	diet	0	10	20	30	40	50	60	70	80	minFREE	0	10	20	30	40	50	60	70	80	minMACRO	0	10	20	30	40	50	60	minADEQ
Proteins	154	154	149	147	141	118	102	94	79	65	74	162	158	152	140	120	103	100	100	100	100	153	150	142	129	123	117	105	126
Carbohydrates	84	84	85	86	89	96	101	103	109	118	109	100	100	100	100	100	103	104	109	109	110	100	100	100	100	100	105	110	101
Fats	189	189	190	191	190	184	182	180	174	161	180	146	149	156	163	174	175	175	164	167	163	145	146	151	162	168	159	154	167
Linoleic acid	220	220	225	242	232	224	260	257	326	414	456	155	171	219	222	221	223	374	358	484	454	203	204	208	257	302	269	321	360
Linolenic acid	127	127	128	131	131	127	129	120	108	145	191	116	116	123	124	126	129	116	108	140	190	122	117	124	135	123	135	146	152
EPA+DHA	140	140	147	180	386	133	104	99	29	11	6	139	146	149	145	133	104	109	131	74	38	239	244	231	229	270	284	242	336
PUFA	106	106	108	116	116	107	121	119	145	185	206	78	85	105	106	106	106	168	161	215	206	100	100	102	123	142	129	151	170
Ca	93	93	95	96	96	91	77	72	51	47	41	96	98	95	90	90	79	76	55	46	41	100	100	100	100	100	100	100	100
Cu	98	98	98	97	89	87	86	83	73	70	79	98	98	97	89	86	88	82	75	81	76	150	150	150	145	145	148	149	101
Fe	68	68	68	66	66	61	57	60	67	73	86	73	73	70	68	60	60	60	72	87	89	100	100	100	102	100	110	136	153
Fibre	56	56	57	58	61	61	61	59	59	54	57	59	60	61	63	62	62	58	56	54	61	100	100	100	100	100	100	100	100
Iodine	80	80	85	87	90	75	66	68	63	58	30	82	84	88	85	74	68	72	75	61	36	100	100	100	100	100	100	100	100
Mg	81	81	74	71	68	62	57	55	50	49	91	81	80	70	66	62	58	57	56	84	95	108	106	102	106	103	103	120	150
P	150	150	149	148	144	126	111	98	74	62	110	154	154	149	140	126	112	104	90	103	125	176	175	175	166	163	157	147	199
K	88	88	79	78	75	68	64	55	45	37	36	88	87	77	73	67	64	57	46	39	41	106	100	100	100	100	100	100	100
Se	194	194	196	219	234	216	211	205	187	177	186	189	192	212	214	210	211	212	242	330	216	193	189	217	219	248	240	208	230
Vitamin A	178	178	182	171	154	143	118	84	64	32	9	165	172	170	151	134	118	76	57	26	9	170	161	171	162	195	173	136	147
Thiamin	100	100	100	98	95	83	72	68	56	47	95	101	101	98	95	82	72	69	64	68	100	114	115	114	111	103	100	100	143
Riboflavin	108	108	108	105	98	83	73	64	40	29	40	110	113	107	96	84	73	67	46	37	42	126	121	120	115	107	100	100	100
Niacin	152	152	140	128	120	95	82	74	60	60	70	154	146	131	117	95	82	77	67	104	80	182	185	175	159	147	150	151	150
Vitamin B_12_	376	376	362	362	408	294	268	239	186	27	18	378	365	352	315	294	268	266	192	145	123	246	236	225	203	326	293	176	228
Pantothenic acid	98	98	99	97	91	73	65	58	48	38	39	99	104	97	89	73	65	59	50	43	45	122	118	118	113	104	100	100	100
Vitamin B_6_	104	104	101	97	96	79	70	63	56	47	78	102	99	94	89	78	71	64	54	60	78	136	137	135	130	122	124	125	150
Folic acid	87	87	86	87	82	75	66	58	53	36	29	91	93	90	82	74	67	58	51	42	33	108	103	109	104	100	100	100	102
Vitamin C	89	89	88	88	82	80	78	50	43	27	12	86	86	86	77	79	78	50	33	15	11	100	100	100	100	100	100	102	100
Vitamin D	70	70	76	86	161	61	47	42	15	13	9	68	74	74	72	60	47	45	35	16	7	100	100	100	100	100	100	100	100
Vitamin E	104	104	107	122	127	132	157	163	215	250	232	75	86	93	105	123	135	198	197	235	223	111	109	119	129	183	131	143	191
Zn	88	88	79	78	73	65	58	54	52	52	64	91	83	80	73	65	59	57	64	74	75	103	100	100	100	100	100	106	123
Cholesterol*	100	100	117	117	110	67	41	36	26	6	3	92	110	116	108	68	41	37	34	12	7	100	100	100	93	67	48	18	16
Free sugars*	99	99	99	99	98	118	120	124	109	195	258	131	126	126	119	120	122	122	106	177	197	100	100	100	99	100	95	97	100
Na*	119	119	120	124	139	122	119	120	101	105	53	138	141	139	136	126	121	121	130	108	81	100	100	100	100	100	100	100	81
SFA*	143	143	142	123	120	111	100	105	94	77	82	113	113	111	109	108	101	102	91	76	60	100	100	100	100	100	100	99	87

FREE; no nutritional constraints; MACRO, constraints on macronutrients only; ADEQ, constraints on all nutrients; GHGE, greenhouse gas emissions; minFREE, maximal GHGE reduction (82·6 %) achievable under the constraints of the FREE scenario; minMACRO, maximal GHGE reduction (82·2 %) achievable under the constraints of the MACRO scenario; minADEQ, maximal GHGE reduction (69·7 %) achievable under the constraints of the ADEQ scenario. Results are percentage of the RDA unless otherwise stated.

* Percentage of the upper limit.

In the FREE scenario, the MAR and SED of the modelled diets remained similar to those of the mean observed diet for moderate GHGE reductions (≤30 %). Strengthening the GHGE constraint for reductions higher than 30 % induced a progressive decrease in the MAR and an increase in the SED.

In the MACRO scenario, adding constraints on macronutrients with no imposed reduction of GHGE did not affect the MAR or SED, but increased the MER, when compared with the mean observed diet. When imposing the GHGE constraint, the modelled diets had a similar MAR and SED as in the FREE scenario, whatever the GHGE reductions. For moderate GHGE reductions (≤30 %), the MER of MACRO models were higher than for diets modelled under the FREE scenario (Fig. 1(b)). Strengthening the GHGE reduction tended to reduce the MER.

In the ADEQ scenario, the nutritional constraints ensured that the MAR reached 100 % and the MER 0 %. The nutritional constraints also induced a reduction of SED compared with both the observed diet and the diets modelled under the FREE and MACRO scenarios. This reduction of the SED reflects an increase in total diet weight induced by the set of nutritional constraints. Imposing GHGE reductions up to 30 % did not induce any additional modifications of the SED, but higher GHGE reductions required decreasing the total diet weight, and thus increasing the SED.

### Impact of reduction in greenhouse gas emissions on diet composition

**Fig. 2:**
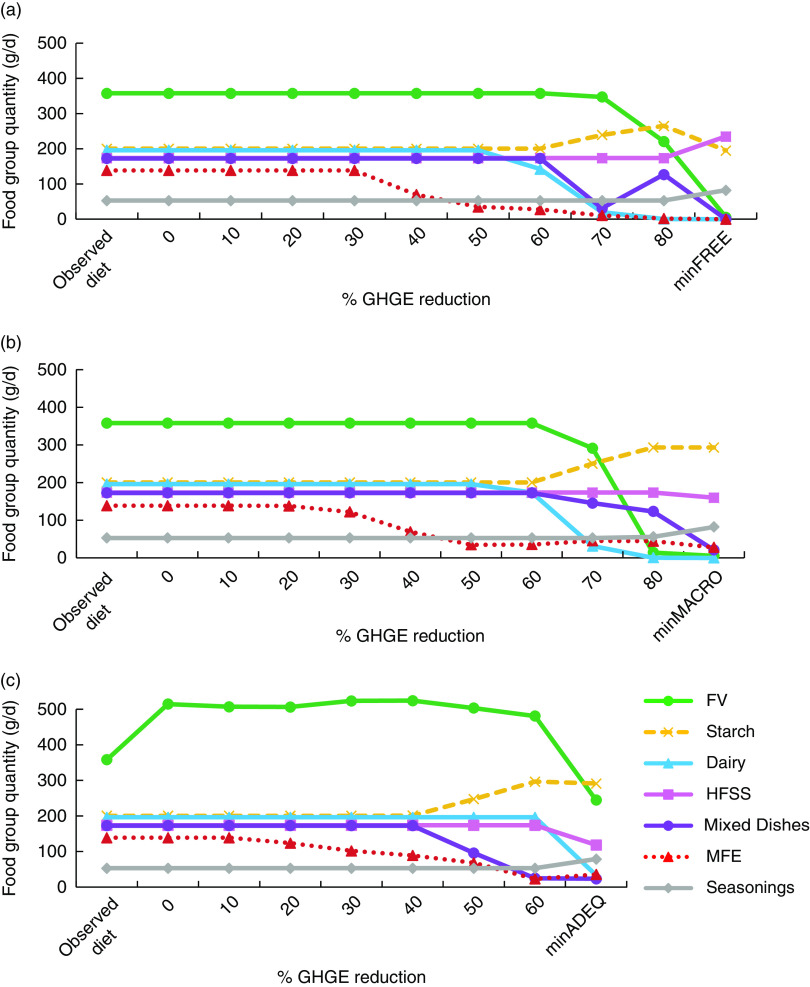
Food group quantities (g/d) in the mean observed diet and at different levels of dietary GHGE reduction from the value of the mean observed diet under the FREE (a), MACRO (b) and ADEQ (c) modelled diets for French women (GHGE, greenhouse gas emissions; FREE; no nutritional constraints; MACRO, constraints on macronutrients only; ADEQ, constraints on all nutrients; FV, Fruits and Vegetables; Dairy, Dairy Products; HFSS, Foods High in Fat/Sugar/Salt; MFE, Meat/Fish/Eggs; minFREE, maximal GHGE reduction (82·6 %) achievable under the constraints of the FREE scenario; minMACRO, maximal GHGE reduction (82·2 %) achievable under the constraints of the MACRO scenario; minADEQ, maximal GHGE reduction (69·7 %) achievable under the constraints of the ADEQ scenario)

The food group quantities of each modelled diet are presented in Fig. 2. In the FREE and MACRO scenarios (Fig. 2(a) and 2(b)), food group quantities did not deviate from those of the mean observed diet for moderate GHGE reductions (≤30 %). Reductions ranging from 30 to 60 % induced a progressive decrease in the Meat/Fish/Eggs (MFE) group quantities. Higher GHGE reductions (≥60 %) required additional food group modifications, namely an increase of the Starch group quantities and a reduction in quantities of the Fruits and Vegetables (FV) and Dairy Products (Dairy) groups. The Seasonings and Foods High in Fat/Salt/Sugar (HFSS) groups did not deviate from their quantity in the mean observed diet, whatever the strength of the GHGE constraint.

**Fig. 3:**
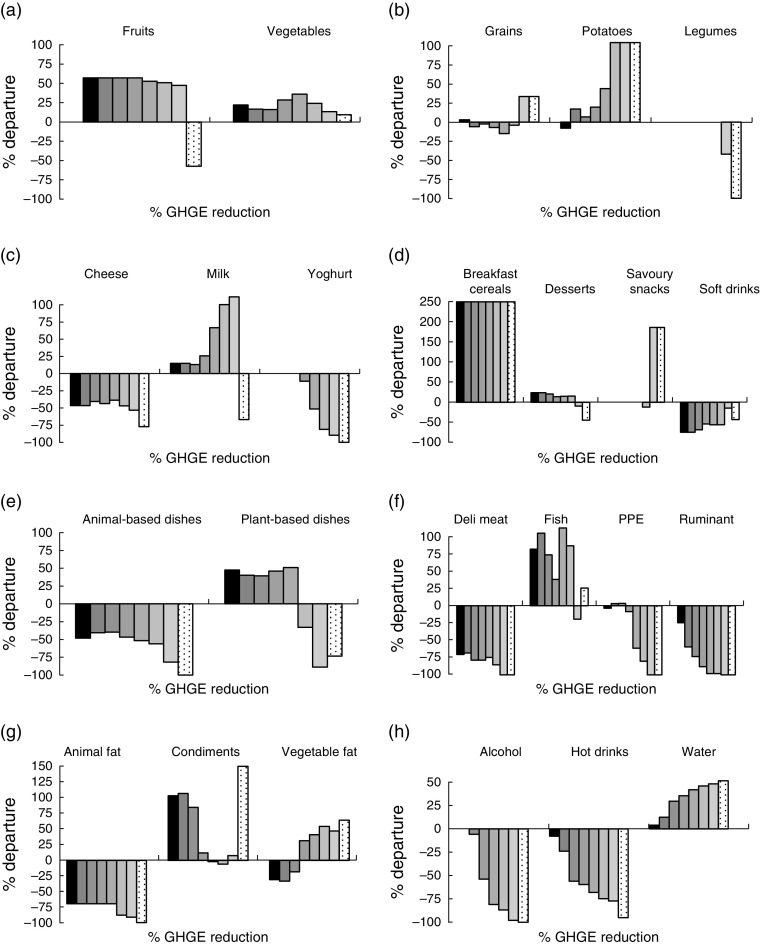
Departure from subgroup quantities in the observed diet (%) for the ADEQ scenario at different levels of dietary GHGE reduction (% GHGE reduction: 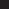, 0; 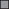, 10; 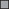, 20; 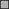, 30; 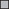, 40; 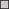, 50; 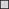, 60; 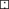, min) for French women, according to food group: (a) Fruits and Vegetables; (b) Starch; (c) Dairy Products; (d) Foods High in Fat/Sugar/Salt; (e) Mixed Dishes; (f) Meat/Fish/Eggs; (g) Seasonings; (h) Drinks (ADEQ, constraints on all nutrients; GHGE, greenhouse gas emissions; min: maximal GHGE reduction (69·7 %) achievable under the constraints of the ADEQ scenario; PPE, pork, poultry and eggs)

In the ADEQ scenario, respecting the set of nutritional constraints with no imposed reduction of GHGE increased the FV quantity up to approximately 500 g/d (Fig. 2(c)). This was explained mainly by an increase in fruit quantity (+57 % from the quantity in the observed diet), as shown in Fig. 3(a) presenting the food subgroup quantities. Adding a GHGE constraint up to 40 % reduction did not require any additional changes in food group quantities, except progressively larger decreases in MFE quantities for reductions ≥20 %. However, other substitutions occurred within food groups (Fig. 3). Within the Dairy food group (Fig. 3(c)), cheese was reduced by almost 50 % from its quantity in the observed diet, in favour of milk. Within the Mixed Dishes food group (Fig. 3(e)), mixed dishes containing animal products were approximately halved in favour of plant-based mixed dishes. Within the MFE food group (Fig. 3(f)), deli and ruminant meats were substituted by the fish subgroup. GHGE reductions higher than 40 % were associated with further decrease in the MFE quantities (suppression of ruminant meat and progressive decrease of the pork, poultry and eggs (PPE) subgroup) together with additional changes at the food group level: the Mixed Dishes group quantity decreased (both animal- and plant-based subgroups) and the Starch group quantity increased (mainly higher quantities of grains and potatoes; Fig. 3(b)). Quantity of the HFSS group remained equal to its levels in the observed diet whatever the GHGE reduction.

### Impact of reduction in greenhouse gas emissions on acceptability

In terms of acceptability, the absolute departure from the mean observed diet remained very low for moderate GHGE reductions in the FREE and MACRO scenarios. In the ADEQ scenario, imposing the set of nutritional constraints with no imposed reduction of GHGE induced a departure of approximately 30 % from the observed diet at the food level and of 5 % at the food group level (Fig. 1(e) and 1(f)). For moderate GHGE reductions (≤30 %), the absolute departure remained close to these values. Higher GHGE reductions (>30 %) increased departure from the observed diet significantly.

### Impact of reduction in greenhouse gas emissions on diet cost

The cost of the mean observed diet was 6·4 €/d (Fig. 1(d)). The cost of the diets modelled under the FREE and MACRO scenarios were similar to or lower than the cost of the mean observed diet. Under those scenarios, strengthening the GHGE constraint tended to reduce diet cost.

Imposing the nutritional constraints of the ADEQ scenario slightly increased the cost of the diet. High GHGE reductions (≥50 %) decreased diet cost.

### Minimization of greenhouse gas emissions

Changing the objective function for a minimization of GHGE induced dramatic changes in terms of diet composition (Figs 2 and 3). Minimizing the GHGE induced elimination of the Dairy group for both FREE and MACRO scenarios (Fig. 2(a) and 2(b)). Without any nutritional constraints (FREE scenario), the MFE and Mixed Dishes groups were also eliminated, while FV was reduced to less than 6 g/d, leading to a modelled diet composed mainly of three food groups: HFSS, Starch and Seasonings, with almost half of the energy provided by HFSS. In the ADEQ scenario, minimizing the GHGE did not suppress any food category, but induced reductions of the FV quantity down to 245 g/d and of MFE down to 35 g/d.

### Strength of nutritional constraints

The comparative strength of each nutritional constraint was evaluated through the analysis of dual values (data not shown). In the MACRO scenario, the most difficult constraint to meet was the minimum energy contribution of carbohydrates (≥50 %) for GHGE reductions ≤50 % and of proteins (≥10 %) for highest GHGE reductions. In the ADEQ scenario, the minimum quantity of fibre (>30 g) was the most difficult constraint to meet. Respecting the upper limit of Na (<2365 mg) was also constraining, especially for GHGE reductions <40 %. Strengthening of the GHGE constraint increased the difficulty in meeting the recommendations for Ca and K. These two recommendations were the most difficult to meet, followed by fibre, when GHGE was minimized.

The main impacts of GHGE reductions on the nutritional quality, diet composition and diet cost observed for women were similar for men’s models. These are presented in the online supplementary material, Supplemental Table 2 and Supplemental Figs 1–3.

## Discussion

Based on three nationally representative French data sets, namely on dietary intakes, food prices and food GHGE, the present study shows that diet-related GHGE may be reduced by 30 % while reaching nutritional adequacy without requiring major additional dietary shifts, at the food group level, than those induced by meeting nutritional recommendations, and at a similar cost. Combining cultural acceptability and nutritional adequacy at higher GHGE reductions (>30 %) was not achievable. Such GHGE reductions either impaired nutritional quality, even when recommendations on macronutrients were imposed, or required non-trivial dietary shifts compromising acceptability to reach nutritional adequacy.

By modelling diets at increasingly stringent levels of nutritional constraints, the present study shows that the nutritional dimension of diet sustainability should not be overlooked when acting on GHGE mitigation. Imposing constraints on macronutrients only (proteins, lipids and carbohydrates) did not ensure nutritional adequacy any better than with no nutritional constraint, and even impaired it: the MER actually increased for moderate GHGE reductions (≤30 %), indicating higher quantities of nutrients to be limited, namely free sugars and Na. Imposing higher GHGE reductions (>30 %) without stringent nutritional constraints decreased the MAR, indicating a diet with lower quantities of beneficial nutrients. Hence in the absence of constraints imposing nutritional adequacy, nutritional quality was impaired by GHGE reduction. These results highlight the need to consider the nutritional dimension through appropriate indicators when investigating alternative diets with lower environmental impact.

By modelling diets minimizing the GHGE, the present study showed that the maximal GHGE reduction achievable from the observed level, while respecting all the nutritional recommendations, was 69·7 % for women and 74·0 % for men. A similar level of GHGE reduction (70 %) was investigated by Audsley *et al*. through a combination of mitigation measures from different sectors^(^ ^6^ ^)^. Sáez-Almendros *et al.* also suggested that a shift towards a Mediterranean diet would result in a 72 % reduction of the Spanish GHGE^(^ ^41^ ^)^, but this required extreme energy restrictions. Our results showed that reaching the maximal reductions of 69·7 % and 74·0 % without energy restrictions required extreme food pattern changes and departure from the observed diet, thus compromising the acceptability of such alternative diets. The model could not identify a combination of foods respecting all the constraints for higher GHGE reductions (>70 %), indicating that nutritional adequacy could not be reached with habitual food items for such a level of GHGE reduction. The nutrients for which needs were the most difficult to fulfil for high GHGE reductions were fibre, Ca and K.

By modelling diets at increasingly stringent levels of nutritional and GHGE constraints, the present study reveals that moderate GHGE reductions did not require any dietary shifts at the food group level additional to those induced by meeting nutritional recommendations, i.e. mainly an increase in fruits and vegetables. This indicates that adopting a nutritionally adequate diet could be coupled to moderate GHGE reductions, and thus would benefit both the environmental and the nutritional dimensions of diet sustainability. However, reaching nutritional adequacy at higher GHGE reductions required further dietary shifts, namely a reduction of foods of animal origin (except fish products), and especially deli and ruminant meats, from 20 % GHGE reduction. The progressive reduction of meat products when the GHGE constraint was strengthened confirmed the role of animal-based foods as the main levers to reduce diet-related GHGE^(^ ^5^ ^,^ ^42^ ^,^ ^43^ ^)^. GHGE reductions higher than 40 % required an increase in quantity of the starchy food group, which was the main component (approximately one-third of the total energy) of the nutritionally adequate diet minimized on GHGE. Moreover, without adequate constraints on micronutrients, high GHGE reductions resulted in the elimination of some food groups, namely Dairy and MFE. Conversely, all food groups were represented in nutritionally adequate diets with reduced GHGE (ADEQ), even when GHGE were minimized. Hence while vegetarian or vegan diets are often claimed to reduce the environmental impact of diet, the results of the present study suggest that food group diversity must be preserved to improve diet sustainability, rather than drastic dietary changes excluding food categories. Previous studies have estimated that shifting from the average diet to a vegetarian diet would reduce GHGE by 22 % in the UK^(^ ^44^ ^)^ or 27 % in Denmark^(^ ^45^ ^)^. However, the realism of such scenarios is questionable since the prevalence of vegetarianism is quite low in industrialized countries (e.g. estimated to be approximately 2 % in the French^(^ ^46^ ^)^ and US^(^ ^47^ ^)^ populations). In addition, some studies suggest that a large proportion of the population is not yet ready to consume a fully plant-based diet^(^ ^48^ ^,^ ^49^ ^)^. According to the present results, 30 % GHGE reduction could be achieved in a nutritionally adequate diet by increasing fruits and vegetables while maintaining intake of meat/fish/eggs at approximately 100 g/d, mainly by substituting ruminant and deli meats by fish products.

The main strength of the present study was taking into account simultaneously several dimensions of diet sustainability, namely nutritional adequacy, environmental impact, affordability and cultural acceptability. The latter was considered by minimizing the departure from the observed diet and through constraints applied on food quantities based on the most recent dietary survey conducted in the French population. Also, whereas previous diet modelling studies were based on eighty-two foods in the UK^(^ ^22^ ^)^ and seventy-six foods items in New Zealand^(^ ^23^ ^)^, the diets modelled in the present study were based on consumption, nutritional composition, GHGE and price of 402 foods identified among those most consumed by the INCA2 participants and representing the consumption of the 1342 foods declared in the national survey.

A further strength of the study was the reliability of the environmental data. Whereas most of the studies assessing the environmental impact of diet are based on environmental data compiled from heterogeneous studies conducted under different LCA modelling hypotheses or specific in terms of geographical situation or production modes, the present study was based on GHGE data built from a hybrid input–output/LCA standardized method applied to the 402 food items, thus ensuring reliably sourced data representative of national food consumption and production modes^(^ ^32^ ^)^.

However, a limitation of the present study was that nutritional adequacy may have been compromised by varying bioavailability of some key nutrients for which animal sources are known to be more favourable^(^ ^50^ ^–^ ^52^ ^)^. In this context, Fe has been highlighted as of particular concern since animal products are the only source of haem Fe, the most bioavailable source of Fe. Hence further improvements of the models could be achieved by taking the bioavailability of such nutrients into account. Also, other indicators of the environmental impact of diet should be considered. Food production has been shown to account for most of the global water footprint^(^ ^53^ ^)^ and agricultural production, being an integral part of many ecosystems, can restrict or promote their biodiversity, resilience and socio-economic functions. In particular, biodiversity is of great concern in fish production. Future modelling studies could thus benefit from including additional environmental indicators, but the main obstacles to such improvements are the limited availability and access to data at the food level. This study could also be further improved by using an individual diet modelling approach to integrate individual food preferences. Moreover, in our study, acceptability was taken into account by minimizing the departure from the observed diet in terms of food and food group contents, in order to limit the deterioration of cultural acceptability induced by the modelling. However, such method cannot guarantee that the proposed shift modelled by the linear programming would be acceptable to the consumer. In particular, it is a strong, and perhaps unjustified, assumption that departing the least from the mean diet, in terms of food groups defined on a nutritional basis rather than on practical and/or monetary ones, will be more acceptable to the consumer. Some recent modelling studies considered the acceptability dimension by introducing price elasticity and food expenditure shares in the model to better control the level and type of deviation from current diet^(^ ^54^ ^)^. However, incorporating information on consumer behaviour still does not necessarily ensure that dietary scenarios would be fully acceptable^(^ ^55^ ^)^. This emphasizes the importance of coupling the identification of more sustainable diets with studies on interventions and tools aimed at favouring their adoption by consumers in real life.

## Conclusion

The current modelling study highlights the need to consider the nutritional dimension through relevant indicators when assessing how to improve diet sustainability. It also shows that nutritional adequacy, cultural acceptability and affordability of the diet may not be compatible with GHGE reductions higher than 30 %. This underlines the limits of the food consumption shift strategy to reduce GHGE and emphasizes that reaching GHGE reduction targets requires to combine mitigation approaches from different sectors, including production efficiency, demand restraint and food system transformation^(^ ^56^ ^)^.

## Supplementary material

For supplementary material accompanying this paper visit https://doi.org/10.1017/S1368980016000653. click here to view supplementary material
