# Supplementary material for: How low can dietary greenhouse gas emissions be reduced without impairing nutritional adequacy, affordability and acceptability of the diet? A modelling study to guide sustainable food choices
Source: Public Health Nutr. 2016 Apr 6;19(14):2662–74. doi: 10.1017/S1368980016000653 (PMC10448381; doi:10.1017/S1368980016000653)

# Online Supplementary Material

## Supplemental methods

### Aggregation of dietary data

Food consumption data were available across 1,342 food items declared in the INCA2 survey. GHGE data were available for 402 food items selected among those most consumed by INCA2 participants. The consumption data of each food not included in the 402 foods list was reported on a one-to-one basis on the closest food among the 402 food items identified using a nutritional Euclidean distance method. The 1,342 foods were first categorized into 46 food families according to similarities in nutritional and food consumption habits. The nutritional Euclidean distance was then calculated between foods within each of the 46 food categories using the SAS PROC DISTANCE procedure, on the basis of their energy, water, protein, fiber, alcohol, sodium, calcium, iron, zinc, vitamin A, vitamin E, vitamin C, fats and SFA contents.

Energy and nutrient intakes calculated with the original database of 1,342 foods and with the aggregated database of 402 foods are given in **Supplemental Table 1**. The mean intakes calculated from the original and aggregated databases were closely correlated for most nutrients, thus confirming the validity of the Euclidean distance method to report individual food intakes.

## Supplemental Tables and Figures

#### Supplemental Table 1. Mean nutrient (%RDA) and energy (kcal) intakes of French adults (n = 1,918) calculated from original and aggregated food databases

|  | **Original database (1342 foods)** | **Aggregated database**  **(402 foods)** | | **Pearson correlation coefficients^1^** | |
| --- | --- | --- | --- | --- | --- |
| Energy intake (kcal) | 2124 | | 2128 | | 0.99 |
| **Nutrient intakes (%RDA)** |  | |  | |  |
| Proteins | 160.3 | | 161 | | 0.99 |
| Fats | 188 | | 189.1 | | 0.98 |
| Carbohydrates | 86.2 | | 87 | | 0.99 |
| Free sugars | 90.9 | | 95.7 | | 0.98 |
| SFA | 144.2 | | 145 | | 0.98 |
| Cholesterol | 112 | | 114.3 | | 0.96 |
| Linolenic acid | 94 | | 124.5 | | 0.70 |
| Linoleic acid | 198.2 | | 212.2 | | 0.93 |
| PUFA | 93.7 | | 103 | | 0.92 |
| Fiber | 62.2 | | 62.2 | | 0.99 |
| Vitamin B1 | 101.8 | | 103.1 | | 0.78 |
| Vitamin B2 | 111.6 | | 115.6 | | 0.94 |
| Vitamin B3 | 154.8 | | 156.4 | | 0.87 |
| Vitamin B5 | 106.6 | | 112.6 | | 0.94 |
| Vitamin B6 | 105.8 | | 106.8 | | 0.93 |
| Vitamin B12 | 383.9 | | 436.3 | | 0.83 |
| Vitamin B9 | 92.3 | | 89.3 | | 0.95 |
| Vitamin A | 168.1 | | 169.3 | | 0.89 |
| Vitamin D | 88.6 | | 121.6 | | 0.61 |
| Vitamin E | 105.7 | | 107.8 | | 0.92 |
| Vitamin C | 86.1 | | 87.7 | | 0.96 |
| Sodium | 210.3 | | 217.7 | | 0.96 |
| Magnesium | 83.3 | | 82.6 | | 0.92 |
| Phosphorus | 170 | | 171.3 | | 0.97 |
| Potassium | 98.8 | | 97.9 | | 0.99 |
| Calcium | 115.2 | | 119.8 | | 0.97 |
| Iron | 110.8 | | 113.3 | | 0.98 |
| Copper | 128.6 | | 133.7 | | 0.89 |
| Zinc | 96.1 | | 93.8 | | 0.96 |
| Selenium | 181 | | 186.4 | | 0.92 |
| Iodine | 88.6 | | 87.4 | | 0.93 |

^1^Pearson correlations between intakes calculated from the original and aggregated databases in n = 1,918 adults. PUFA: polyunsaturated fatty acids; SFA: saturated fatty acids

#### Supplemental Table 2. Nutrient levels in the observed diet and the FREE, MACRO and ADEQ modeled diets at different levels of GHGE reduction, for men

|  |  | **FREE** | | | | | | | | | | **MACRO** | | | | | | | | | **ADEQ** | | | | | | | | |
| --- | --- | --- | --- | --- | --- | --- | --- | --- | --- | --- | --- | --- | --- | --- | --- | --- | --- | --- | --- | --- | --- | --- | --- | --- | --- | --- | --- | --- | --- |
|  | **OBSERVED DIET** | **% GHGE reduction** | | | | | | | | | | **% GHGE reduction** | | | | | | | | | **% GHGE reduction** | | | | | | | | |
| Nutrients |  | 0 | 10 | 20 | 30 | 40 | 50 | 60 | 70 | 80 | min  FREE | 0 | 10 | 20 | 30 | 40 | 50 | 60 | 70 | min  MACRO | 0 | 10 | 20 | 30 | 40 | 50 | 60 | 70 | min  ADEQ |
| **Proteins** | 156 | 156 | 152 | 156 | 147 | 130 | 114 | 98 | 86 | 73 | 68 | 164 | 162 | 158 | 147 | 131 | 114 | 100 | 100 | 100 | 154 | 157 | 145 | 136 | 118 | 102 | 100 | 100 | 100 |
| **Carbohydrates** | 83 | 83 | 85 | 91 | 94 | 97 | 102 | 106 | 114 | 125 | 115 | 100 | 100 | 100 | 100 | 100 | 102 | 105 | 113 | 112 | 100 | 100 | 100 | 100 | 100 | 100 | 102 | 105 | 106 |
| **Fats** | 175 | 175 | 175 | 176 | 174 | 175 | 173 | 169 | 156 | 139 | 168 | 146 | 146 | 152 | 160 | 168 | 173 | 172 | 155 | 158 | 147 | 147 | 151 | 160 | 172 | 175 | 175 | 173 | 170 |
| **Linoleic acid** | 189 | 189 | 196 | 199 | 200 | 199 | 227 | 247 | 266 | 353 | 388 | 186 | 187 | 194 | 224 | 198 | 227 | 247 | 312 | 387 | 208 | 208 | 208 | 209 | 214 | 248 | 322 | 360 | 350 |
| **Linolenic acid** | 112 | 112 | 113 | 114 | 114 | 115 | 180 | 106 | 95 | 126 | 157 | 109 | 109 | 111 | 115 | 113 | 180 | 106 | 116 | 156 | 113 | 113 | 112 | 109 | 111 | 134 | 126 | 153 | 174 |
| **EPA+DHA** | 147 | 147 | 158 | 159 | 154 | 140 | 132 | 111 | 42 | 14 | 9 | 145 | 149 | 156 | 154 | 140 | 132 | 112 | 139 | 90 | 236 | 233 | 233 | 230 | 233 | 228 | 257 | 253 | 311 |
| **PUFA** | 91 | 91 | 95 | 96 | 96 | 95 | 112 | 113 | 120 | 158 | 175 | 90 | 90 | 93 | 106 | 94 | 112 | 114 | 142 | 176 | 100 | 100 | 100 | 100 | 102 | 118 | 149 | 167 | 165 |
| **Calcium** | 111 | 111 | 114 | 118 | 117 | 116 | 108 | 101 | 74 | 55 | 61 | 110 | 112 | 114 | 118 | 112 | 108 | 101 | 76 | 60 | 116 | 117 | 114 | 100 | 100 | 102 | 100 | 100 | 100 |
| Copper | 92 | 92 | 92 | 85 | 85 | 82 | 80 | 79 | 75 | 68 | 74 | 90 | 90 | 87 | 86 | 81 | 80 | 79 | 76 | 75 | 108 | 128 | 127 | 126 | 115 | 105 | 101 | 100 | 100 |
| **Iron** | 162 | 162 | 160 | 158 | 152 | 141 | 132 | 142 | 163 | 151 | 186 | 167 | 166 | 165 | 155 | 142 | 132 | 141 | 168 | 191 | 165 | 162 | 175 | 193 | 207 | 217 | 228 | 266 | 339 |
| **Fiber** | 69 | 69 | 71 | 74 | 74 | 77 | 76 | 74 | 79 | 74 | 62 | 75 | 75 | 75 | 75 | 77 | 76 | 73 | 79 | 68 | 100 | 100 | 100 | 100 | 100 | 100 | 100 | 100 | 108 |
| **Iodine** | 96 | 96 | 100 | 103 | 103 | 92 | 84 | 87 | 92 | 77 | 45 | 94 | 97 | 102 | 103 | 91 | 84 | 87 | 97 | 77 | 104 | 100 | 105 | 107 | 115 | 114 | 112 | 106 | 119 |
| **Magnesium** | 85 | 85 | 83 | 73 | 70 | 66 | 62 | 63 | 60 | 55 | 80 | 83 | 82 | 73 | 70 | 66 | 62 | 63 | 64 | 87 | 104 | 102 | 100 | 100 | 100 | 100 | 101 | 102 | 143 |
| **Phosphorus** | 195 | 195 | 195 | 195 | 189 | 173 | 156 | 134 | 108 | 91 | 96 | 196 | 196 | 193 | 186 | 170 | 156 | 136 | 123 | 125 | 209 | 213 | 208 | 200 | 190 | 177 | 167 | 166 | 176 |
| **Potassium** | 109 | 109 | 106 | 93 | 89 | 83 | 76 | 69 | 57 | 47 | 39 | 106 | 106 | 91 | 87 | 82 | 76 | 69 | 60 | 47 | 139 | 134 | 125 | 119 | 107 | 105 | 100 | 100 | 100 |
| **Selenium** | 178 | 178 | 181 | 191 | 205 | 201 | 190 | 181 | 166 | 150 | 174 | 183 | 184 | 189 | 200 | 196 | 190 | 182 | 192 | 346 | 180 | 189 | 189 | 218 | 210 | 199 | 191 | 197 | 199 |
| **Vitamin A** | 159 | 159 | 167 | 165 | 151 | 127 | 119 | 72 | 52 | 28 | 9 | 145 | 148 | 152 | 152 | 125 | 119 | 72 | 52 | 14 | 150 | 164 | 167 | 167 | 145 | 114 | 141 | 108 | 133 |
| **Vitamin B1** | 106 | 106 | 107 | 109 | 109 | 98 | 79 | 70 | 64 | 54 | 46 | 108 | 109 | 106 | 102 | 99 | 79 | 71 | 66 | 59 | 118 | 138 | 134 | 130 | 102 | 100 | 100 | 100 | 100 |
| **Vitamin B2** | 124 | 124 | 127 | 119 | 115 | 98 | 86 | 74 | 52 | 38 | 37 | 124 | 126 | 123 | 115 | 98 | 86 | 75 | 54 | 42 | 137 | 135 | 129 | 117 | 105 | 100 | 100 | 100 | 100 |
| **Vitamin B3** | 161 | 161 | 149 | 139 | 126 | 108 | 87 | 75 | 67 | 59 | 63 | 164 | 161 | 144 | 122 | 108 | 87 | 77 | 72 | 106 | 165 | 168 | 159 | 165 | 153 | 139 | 141 | 143 | 138 |
| **Vitamin B12** | 502 | 502 | 480 | 474 | 455 | 401 | 379 | 314 | 86 | 33 | 30 | 502 | 497 | 477 | 454 | 399 | 379 | 317 | 201 | 154 | 345 | 385 | 365 | 532 | 498 | 299 | 277 | 267 | 234 |
| **Vitamin B5** | 128 | 128 | 134 | 120 | 115 | 96 | 87 | 73 | 63 | 53 | 45 | 129 | 131 | 125 | 115 | 97 | 87 | 73 | 64 | 53 | 149 | 145 | 139 | 126 | 116 | 108 | 112 | 100 | 100 |
| **Vitamin B6** | 110 | 110 | 105 | 97 | 92 | 83 | 71 | 62 | 58 | 55 | 39 | 106 | 106 | 99 | 89 | 83 | 71 | 63 | 59 | 46 | 134 | 135 | 133 | 137 | 128 | 122 | 110 | 111 | 114 |
| **Vitamin B9** | 91 | 91 | 95 | 94 | 94 | 85 | 78 | 69 | 60 | 47 | 39 | 96 | 97 | 96 | 93 | 86 | 78 | 68 | 62 | 46 | 100 | 100 | 100 | 100 | 100 | 100 | 100 | 100 | 114 |
| **Vitamin C** | 87 | 87 | 87 | 84 | 84 | 72 | 67 | 52 | 37 | 35 | 17 | 83 | 83 | 81 | 81 | 70 | 67 | 48 | 40 | 17 | 100 | 100 | 100 | 100 | 100 | 100 | 100 | 100 | 100 |
| **Vitamin D** | 76 | 76 | 84 | 83 | 79 | 70 | 61 | 49 | 21 | 10 | 12 | 72 | 74 | 80 | 79 | 68 | 61 | 49 | 43 | 20 | 100 | 100 | 100 | 100 | 100 | 100 | 100 | 100 | 100 |
| **Vitamin E** | 111 | 111 | 114 | 114 | 135 | 146 | 182 | 203 | 237 | 286 | 227 | 97 | 98 | 107 | 114 | 141 | 182 | 202 | 232 | 220 | 111 | 111 | 118 | 156 | 176 | 200 | 253 | 291 | 215 |
| **Zinc** | 100 | 100 | 91 | 92 | 88 | 79 | 69 | 61 | 60 | 54 | 69 | 102 | 99 | 91 | 87 | 78 | 69 | 62 | 68 | 76 | 100 | 100 | 100 | 100 | 100 | 100 | 100 | 100 | 133 |
| **Cholesterol^1^** | 130 | 130 | 158 | 157 | 140 | 94 | 77 | 46 | 27 | 6 | 4 | 120 | 129 | 147 | 138 | 92 | 77 | 48 | 33 | 15 | 100 | 100 | 100 | 100 | 100 | 74 | 38 | 17 | 13 |
| **Free sugars^1^** | 85 | 85 | 85 | 85 | 103 | 101 | 101 | 115 | 104 | 177 | 236 | 118 | 118 | 112 | 114 | 101 | 101 | 111 | 102 | 173 | 100 | 100 | 100 | 100 | 100 | 100 | 100 | 100 | 100 |
| **Sodium^1^** | 136 | 136 | 142 | 155 | 155 | 155 | 148 | 140 | 142 | 136 | 89 | 162 | 162 | 165 | 162 | 160 | 148 | 143 | 150 | 133 | 100 | 100 | 100 | 100 | 100 | 100 | 100 | 100 | 100 |
| **SFA^1^** | 135 | 135 | 134 | 135 | 114 | 110 | 98 | 104 | 87 | 68 | 89 | 107 | 106 | 107 | 109 | 104 | 98 | 106 | 88 | 74 | 100 | 100 | 100 | 100 | 100 | 100 | 100 | 89 | 88 |

Results are percentage of RDA unless stated; **^1^**percentage of upper limit; minFREE: maximal reduction (81.9%) achievable under the constraints of the FREE scenario; minMACRO: maximal reduction (79.9%) achievable under the constraints of the MACRO scenario; minADEQ: maximal reduction (74.0%) achievable under the constraints of the ADEQ scenario; PUFA: polyunsaturated fatty acids; SFA: saturated fatty acids

## Supplemental Figure legends

**Supplemental Figure 1**. Mean adequacy ratio, mean excess ratio, solid energy density, diet cost and departure from the observed diet at different levels of dietary GHGE reduction in the mean observed diet ( ◼ ) and the FREE (🞝🞝▲🞝🞝), MACRO (–◼–) and ADEQ (–•–) modeled diets, for men.

GHGE: greenhouse gas emission; minFREE: maximal reduction (81.9%) achievable under the constraints of the FREE scenario; minMACRO: maximal reduction (79.9%) achievable under the constraints of the MACRO scenario; minADEQ: maximal reduction (74.0%) achievable under the constraints of the ADEQ scenario

**Supplemental Figure 2**. Food group quantities (g/d) at different levels of dietary GHGE reduction in the mean observed diet, FREE (A), MACRO (B) and ADEQ (C) modeled diets for men.

FV: fruits and vegetables; GHGE: greenhouse gas emission; HFSS: foods high in fat/sugar/salt; MFE: meat/fish/eggs; minFREE: maximal reduction (81.9%) achievable under the constraints of the FREE scenario; minMACRO: maximal reduction (79.9%) achievable under the constraints of the MACRO scenario; minADEQ: maximal reduction (74.0%) achievable under the constraints of the ADEQ scenario

**Supplemental Figure 3**. Departure from sub-group quantities in the observed diet (%) for the ADEQ scenario at different levels of dietary GHGE reduction, for men.

GHGE: greenhouse gas emission; HFSS: foods high in fat/sugar/salt; min: maximal reduction (74.0%) achievable under the constraints of the ADEQ scenario.

**Supplemental Figure 1.**


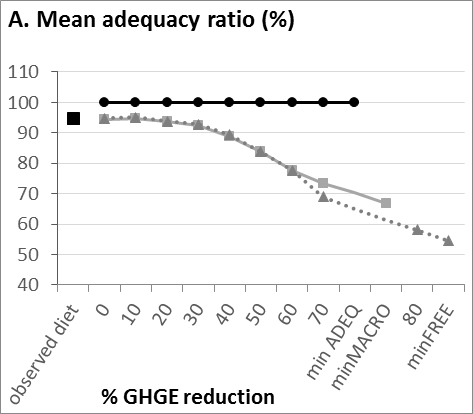

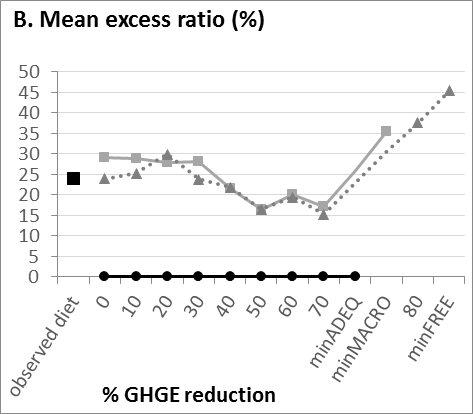

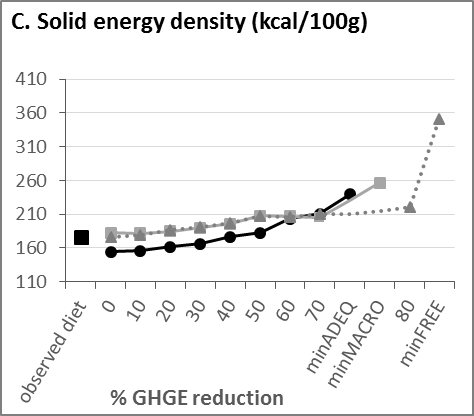

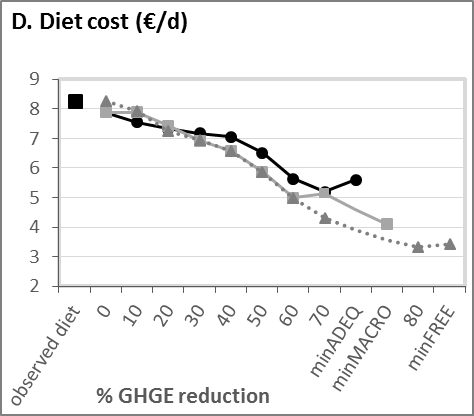

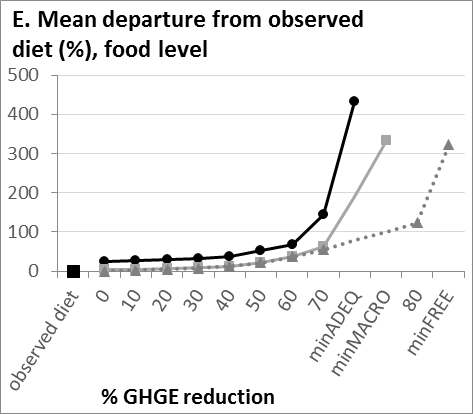

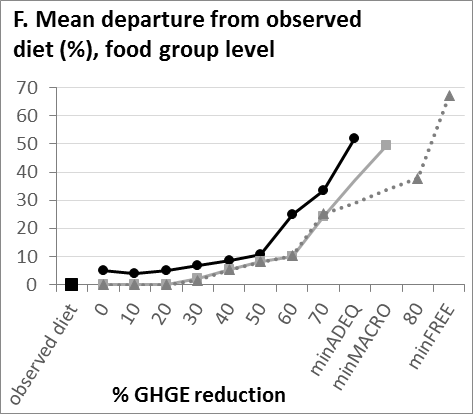


**Supplemental Figure 2.**


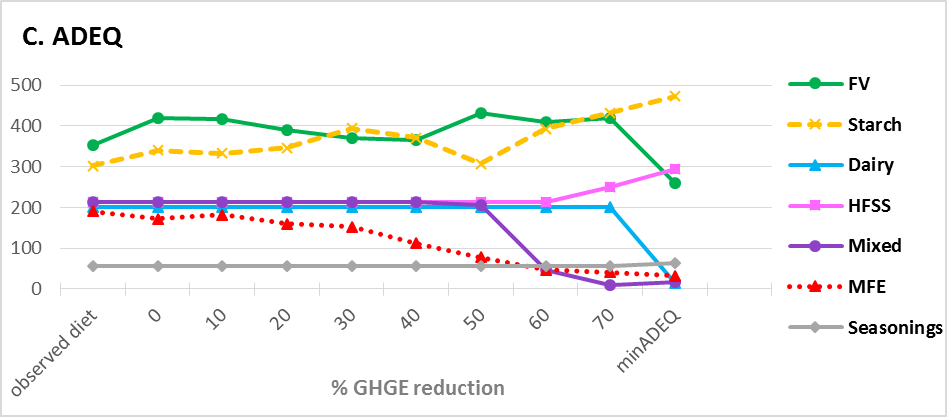

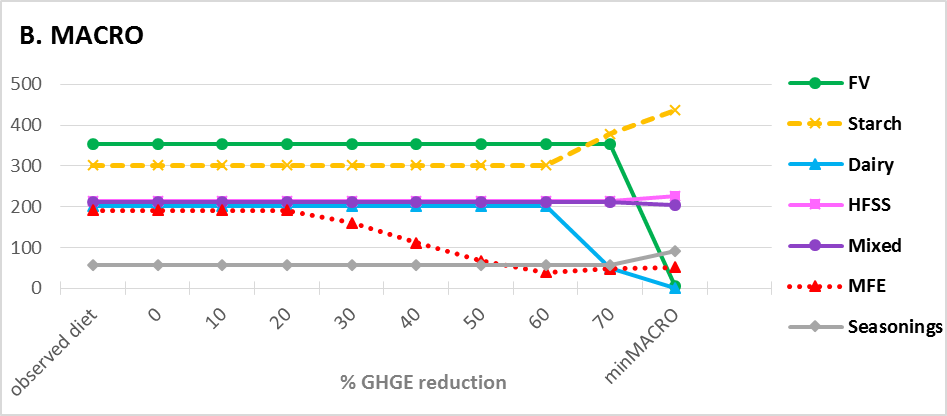

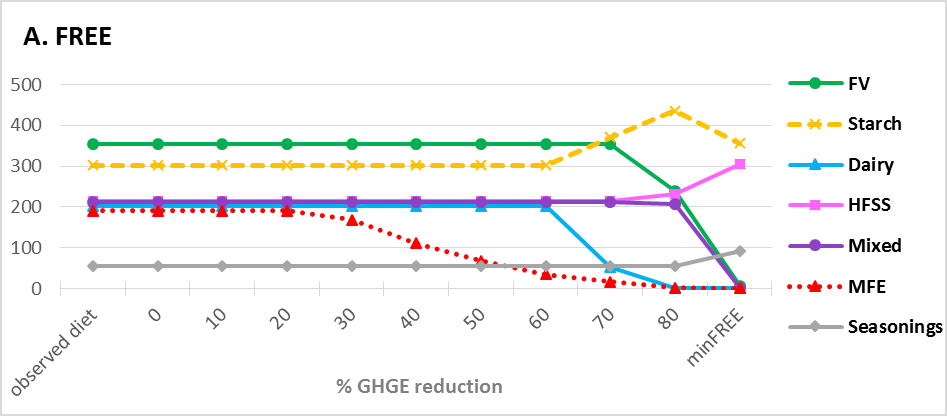


**Supplemental Figure 3.**


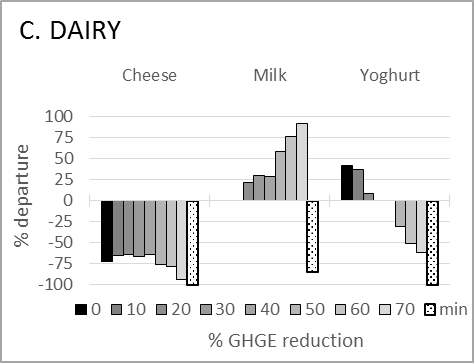

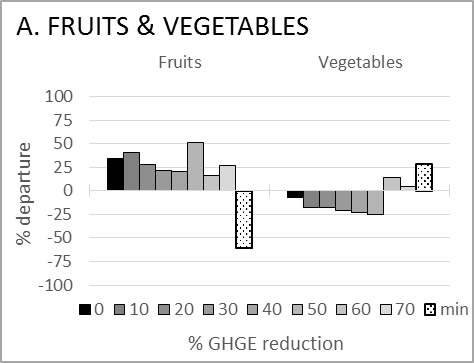

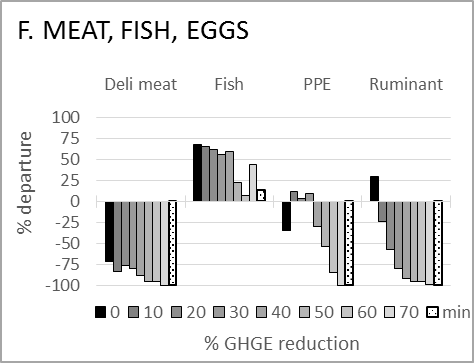

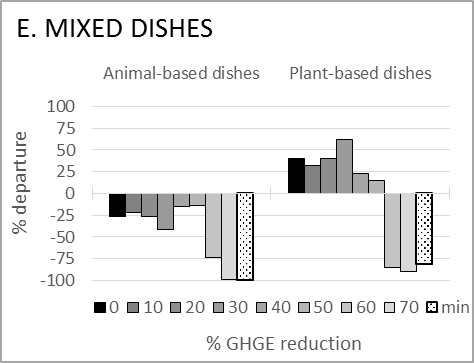

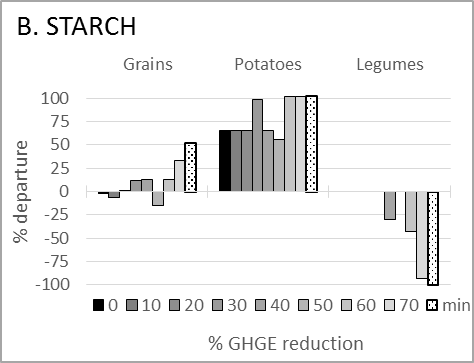

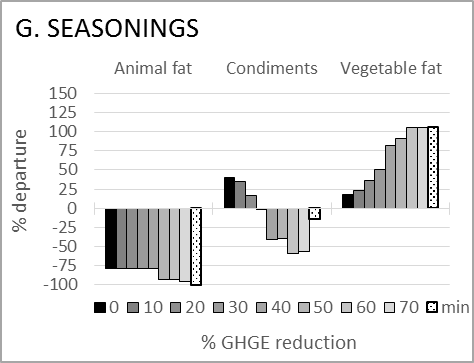

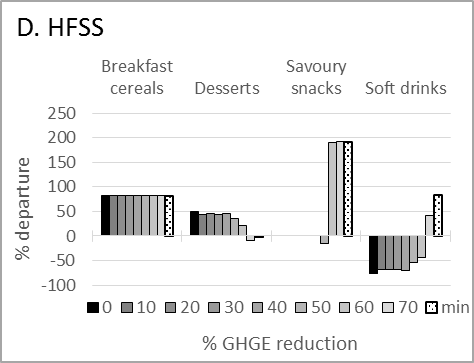

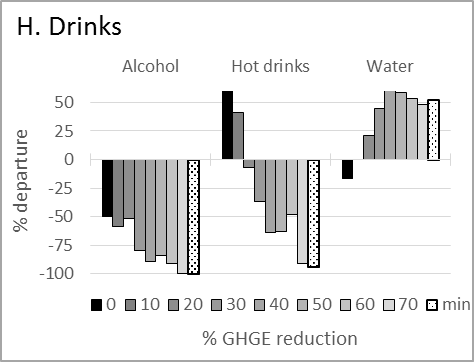

Supplement: Supplementary file 1 [file S1368980016000653sup001.docx]
